# Asymmetry and Allostery: Insights into the Mechanism of Directional Peptide Translocation in AAA+ Unfoldases

**DOI:** 10.64898/2026.07.27.741010

**Published:** 2026-07-29

**Authors:** Tyler G. Southam, Myongin Oh, Jessica M. J. Swanson

## Abstract

Molecular motors convert stochastic ATP chemistry into directional motion. Modern nonequilibrium theories explain this behavior through kinetic asymmetry, yet the molecular mechanisms that generate kinetic asymmetry in AAA+ unfoldases remain unresolved. Cryo-electron microscopy has consistently revealed unfoldases take on an asymmetric helical staircase structure, inspiring the proposed processive hand-over-hand translocation mechanism. In contrast, biochemical and single-molecule studies have reported bursts, slips, and variable step sizes indicative of stochastic behavior. How these observations are reconciled and how directional motion emerges from stochastic ATP hydrolysis remain central questions. Here we use all-atom molecular dynamics simulations of the AAA+ unfoldases Yme1 and Vps4 to explore structural changes induced by hydrolysis, ADP release, and ATP association. The simulations reveal coexisting pre- and post-hydrolysis conformations consistent with experimental cryo- EM densities while also uncovering interconnected allosteric communication networks. One network links the Walker B glutamate to the proximal subunit’s nucleotide binding pocket (NBP), providing a mechanism for backward propagation of hydrolysis competence around the hexamer. Another network couples NBP and salt bridge rearrangements to pore-loop dynamics, connecting subunit position rather than ATP hydrolysis to substrate release. Simulations of nucleotide exchange intermediates further show that ATP binding before ADP and/or Mg ^2+^ release can induce transient strain leading to alternative hydrolysis-competent configurations that still favor forward progression, providing a structural basis for some of the experimentally observed stochastic behavior. Together, these findings suggest that structural asymmetry creates position-dependent allosteric interactions that generate kinetic asymmetry, biasing a fundamentally stochastic ATPase cycle toward directional substrate translocation while still permitting variation in stepping behavior.

**Significance Statement:**

AAA+ ATPases play a central role in biology, converting ATP binding, hydrolysis, and release into the directional forces needed to carry out work, such as substrate translocation, remodeling, and degradation. In addition to being important therapeutic targets, understanding how these molecular machines convert stochastic ATP chemistry into persistent mechanical motion is of fundamental interest. Using all-atom molecular dynamics simulations of Yme1 and Vps4, we identify position-responsive conformational changes and allosteric communication networks that propagate hydrolysis competence, coordinate substrate release, and redistribute the relative probabilities of mechanochemical transitions around the ATPase ring. These findings provide molecular insight into how structural asymmetry and kinetic redistribution enable directional substrate translocation.

## Full Text Availability

The license terms selected by the author(s) for this preprint version do not permit archiving in PMC. The full text is available from the preprint server.

